# Supplementary material for: A direct physical interaction between Nanog and Sox2 regulates embryonic stem cell self-renewal
Source: EMBO J. 2013 Jul 26;32(16):2231–47. doi: 10.1038/emboj.2013.161 (PMC3746198; doi:10.1038/emboj.2013.161)

Figure 1A

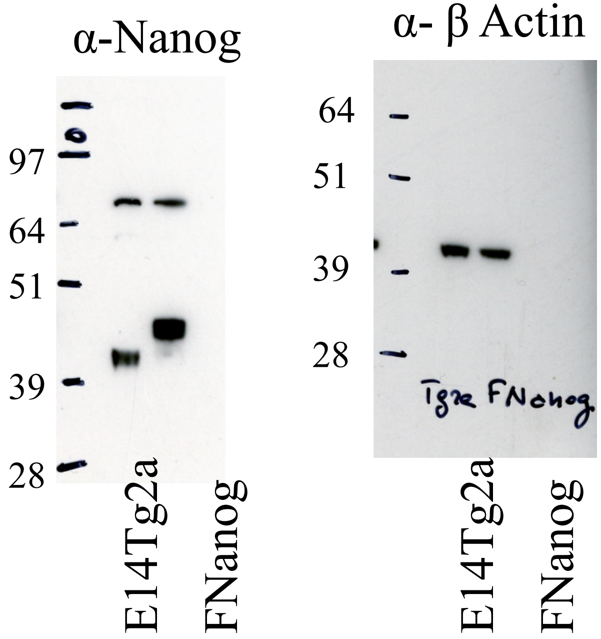

Figure 1E Top

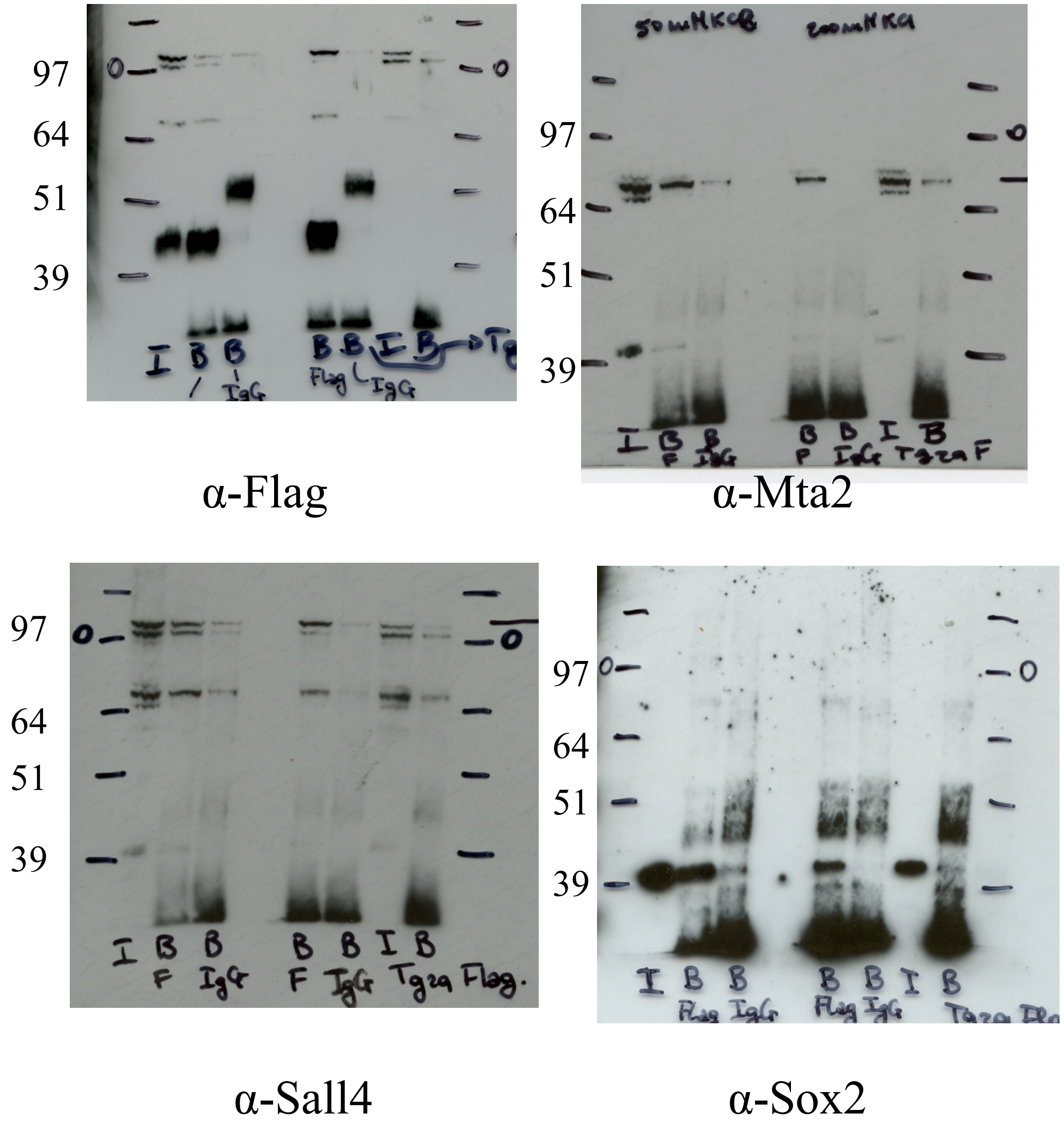

Figure 1E Bottom

Darker immunoblot exposure for PolII IP, FLAG IP and Flag inputs

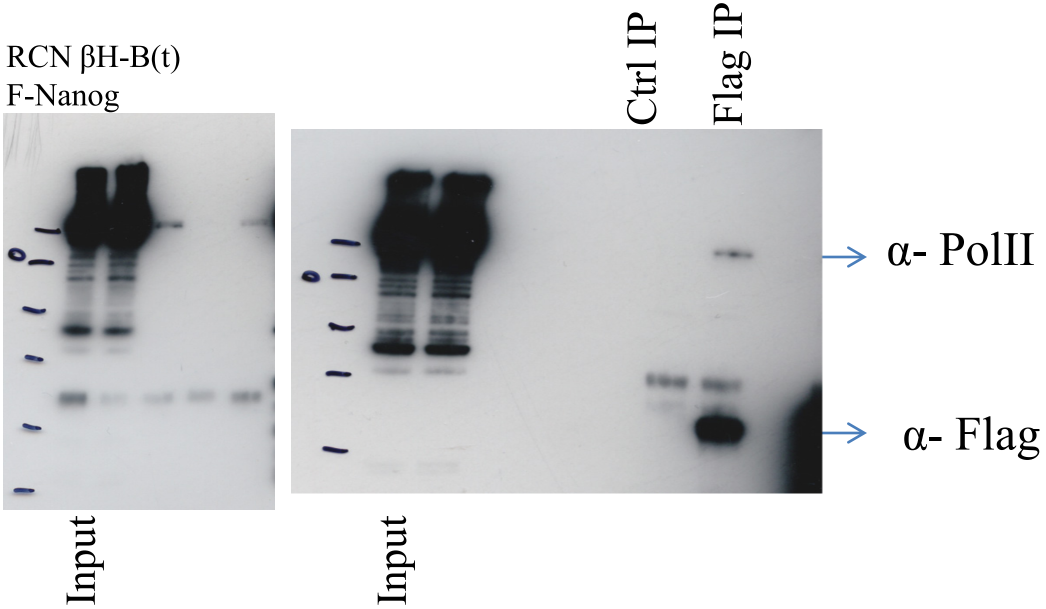

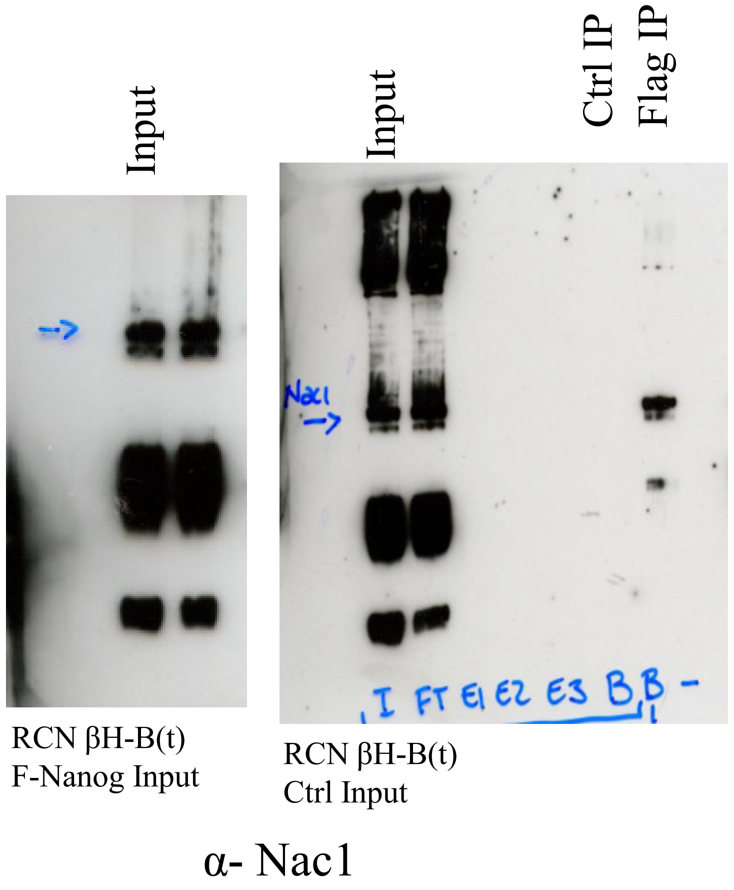

Lighter immunoblot exposure for PolII Inputs

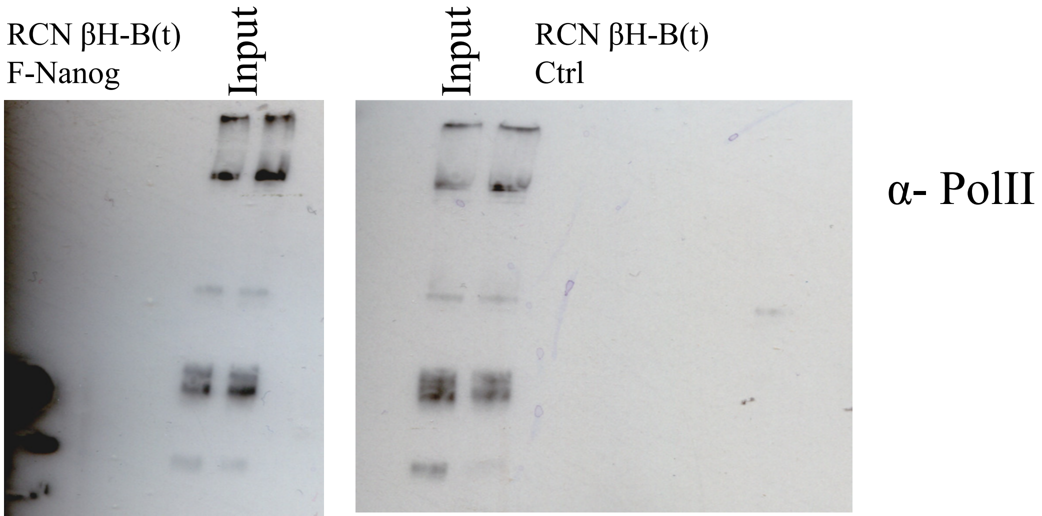

Supplement: Source data for Figure 1 [file emboj2013161df1.pdf]
