# Supplementary material for: A direct physical interaction between Nanog and Sox2 regulates embryonic stem cell self-renewal
Source: EMBO J. 2013 Jul 26;32(16):2231–47. doi: 10.1038/emboj.2013.161 (PMC3746198; doi:10.1038/emboj.2013.161)

Figure 3A

Sox2 IP

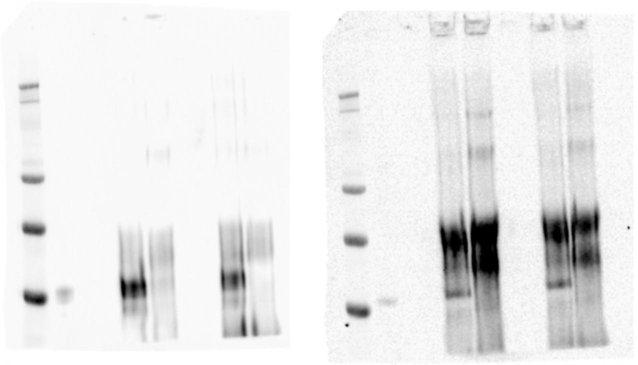

IB: α- Nanog IB: α- Sox2

Figure 3B

Nanog IP

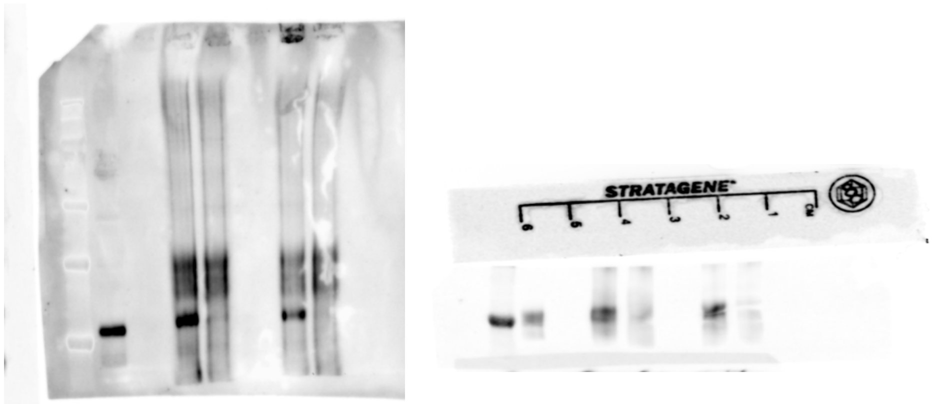

IB: α- Sox2 IB: α- Nanog

Figure 3C

HA IP

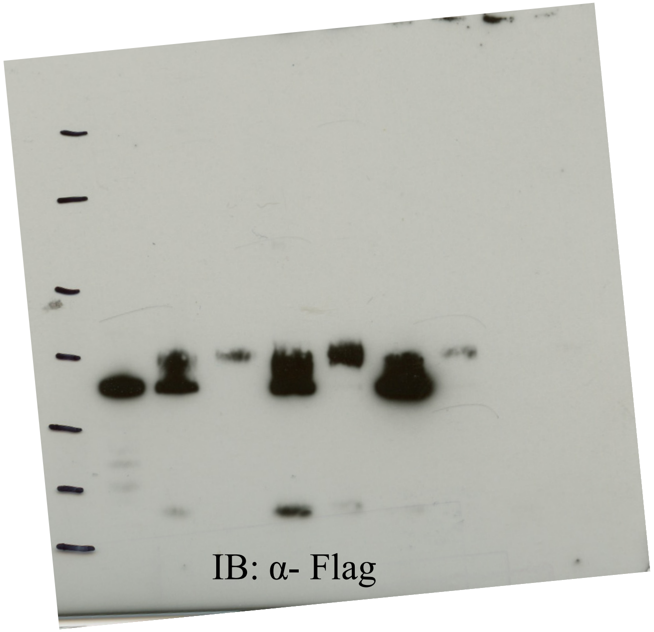

IB: α- Flag

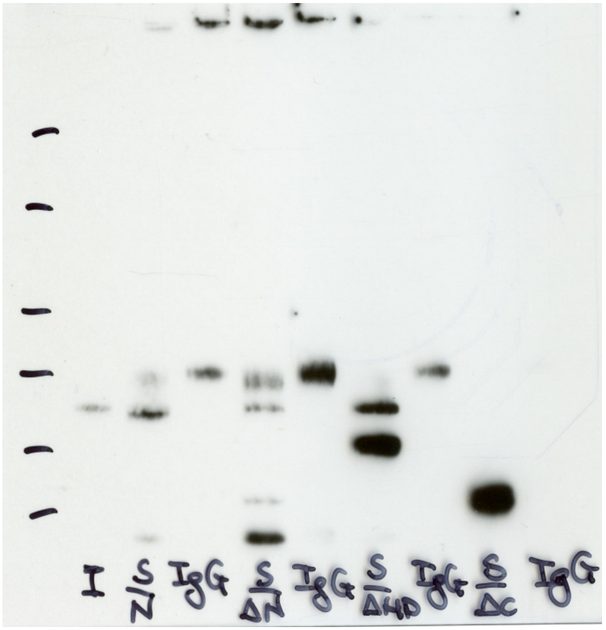

IB: α- HA

Figure 3D

HA IP

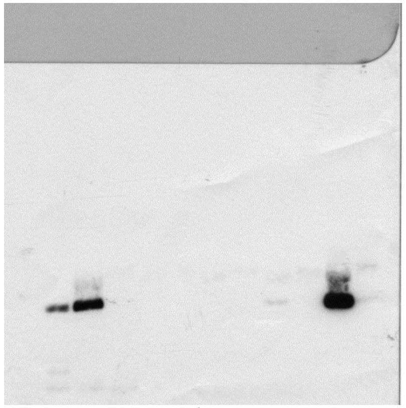

IB: α- Flag

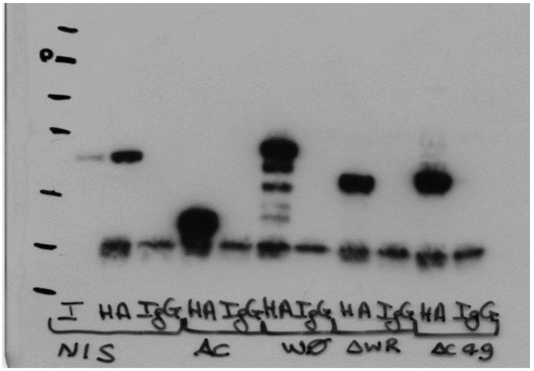

IB: α- HA

Figure 3E
Top

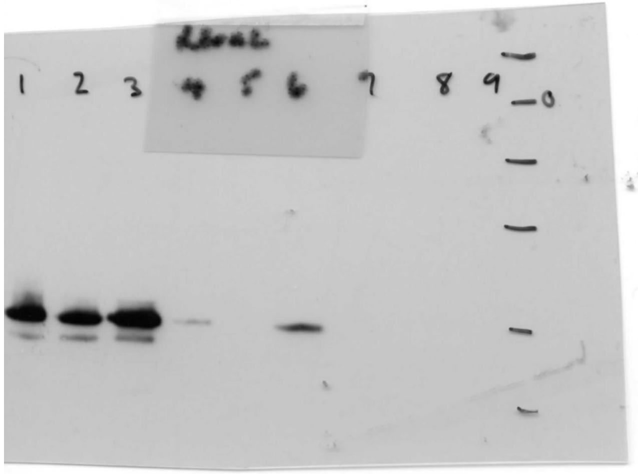

Figure 3E
Bottom

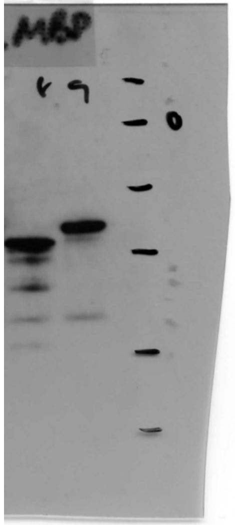

Supplement: Source data for Figure 3 [file emboj2013161df3.pdf]
