# Supplementary figures and images for: A direct physical interaction between Nanog and Sox2 regulates embryonic stem cell self-renewal
Source: EMBO J. 2013 Jul 26;32(16):2231–47. doi: 10.1038/emboj.2013.161 (PMC3746198; doi:10.1038/emboj.2013.161)

Figure 4A

HA IP

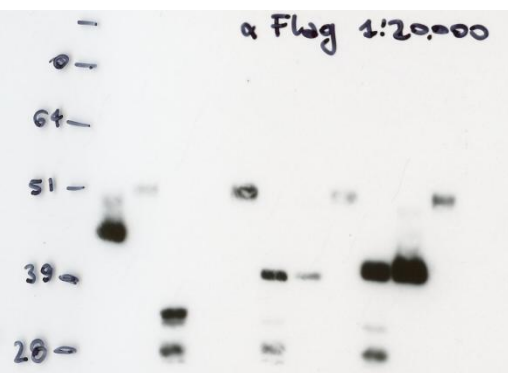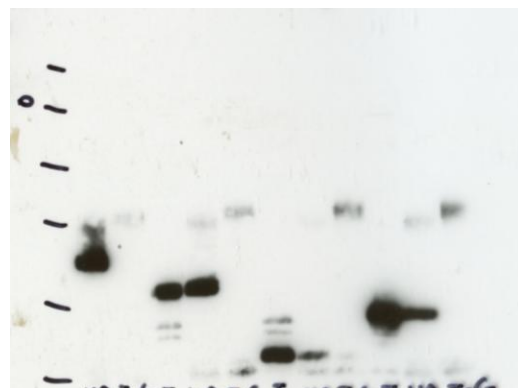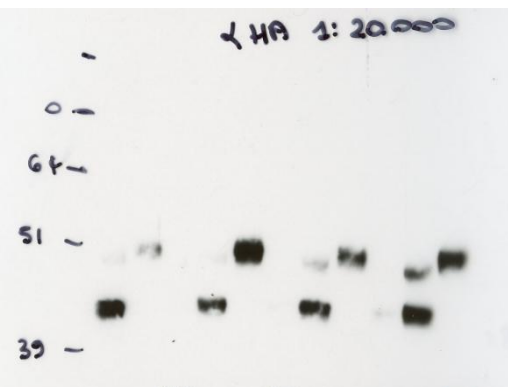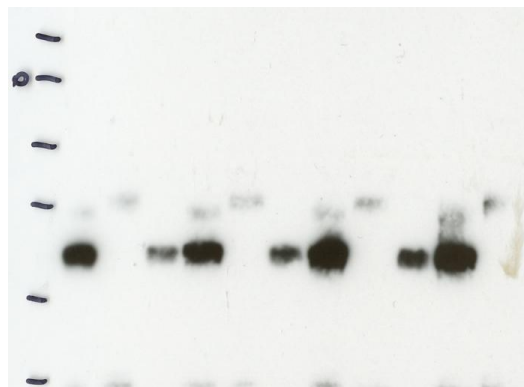

Figure 4B

HA IP

IB:  $\alpha$ - Flag

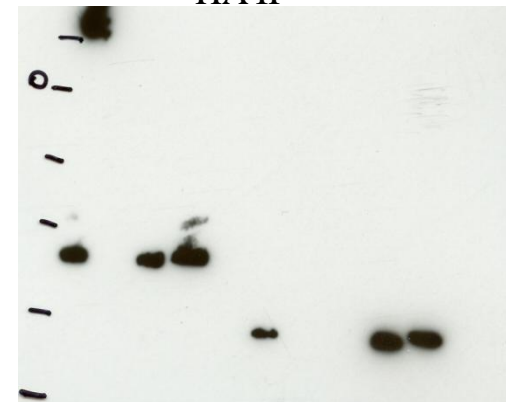

IB:  $\alpha$ - HA

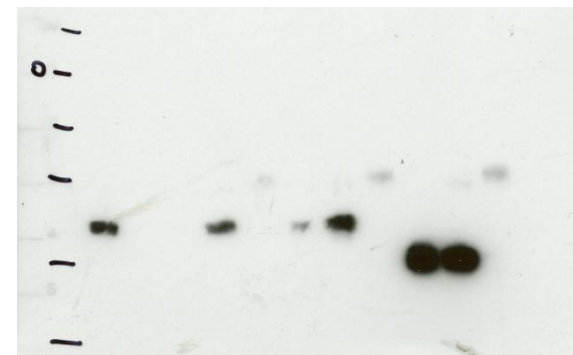

Supplement: Source data for Figure 4 [file emboj2013161df4.pdf]

Figure 5B

HA IP

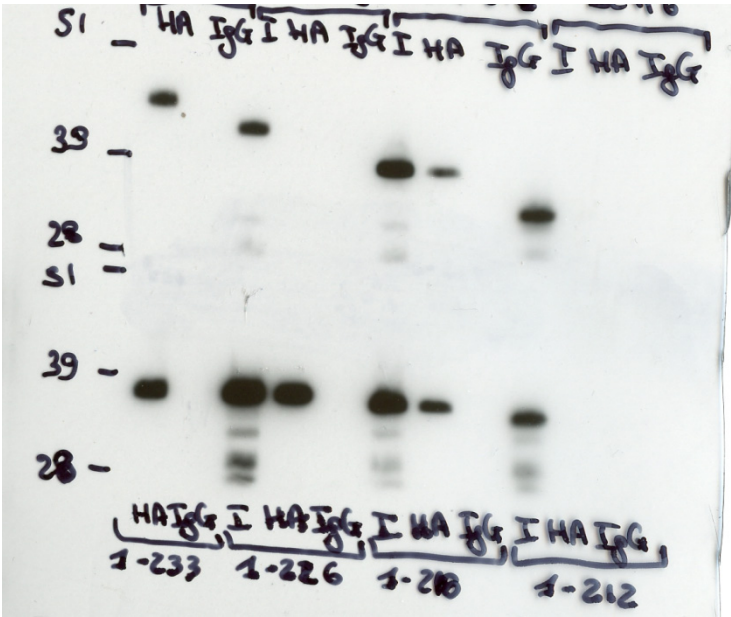

IB: α- Flag

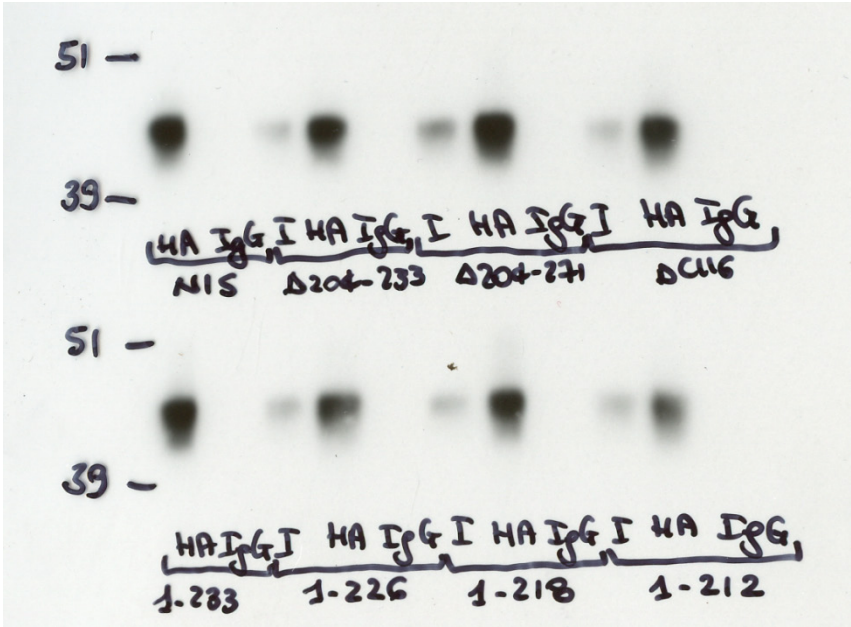

IB: α- HA

Supplement: Source data for Figure 5 [file emboj2013161df5.pdf]

Figure 8D

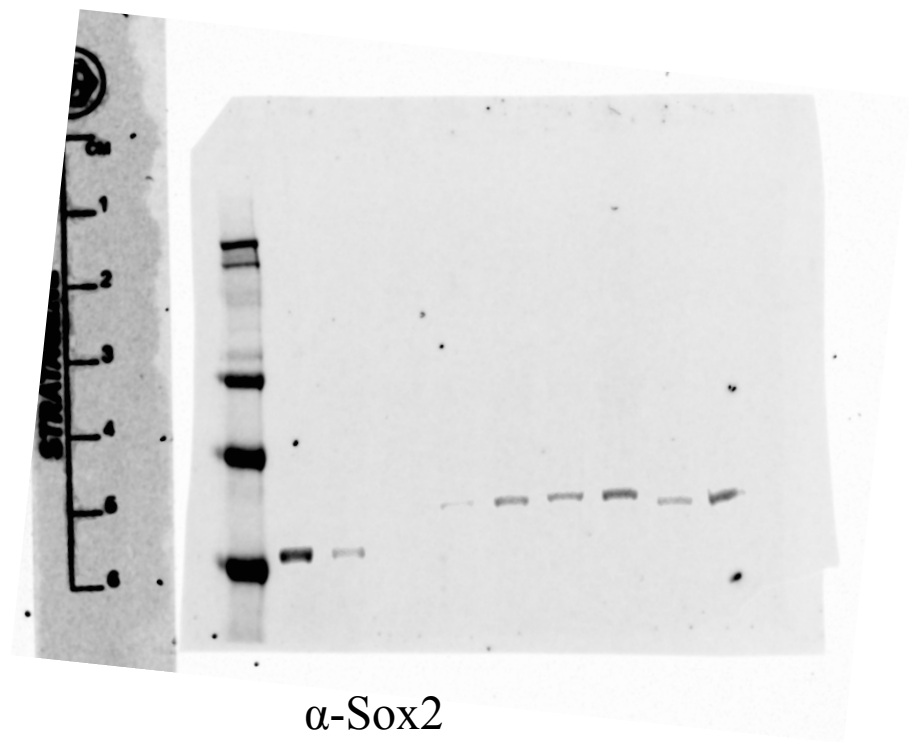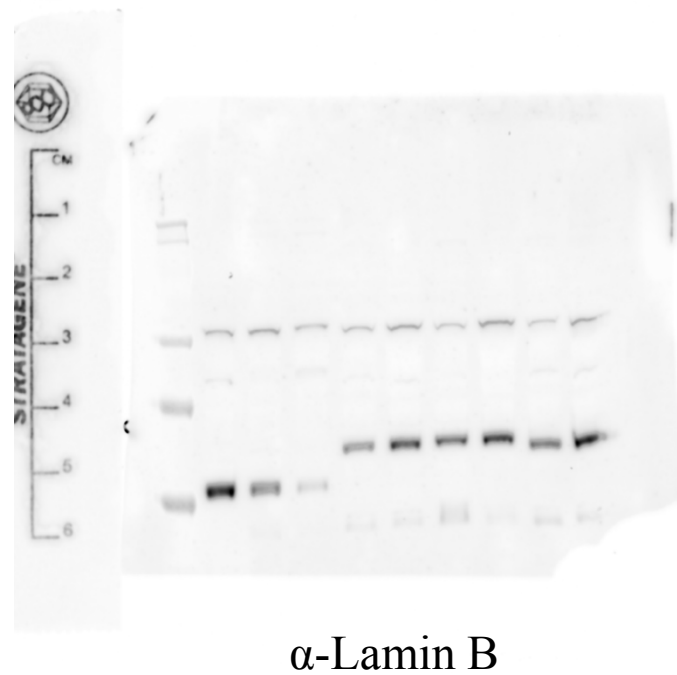

Supplement: Source data for Figure 8 [file emboj2013161df8.pdf]
