# Supplementary material for: A direct physical interaction between Nanog and Sox2 regulates embryonic stem cell self-renewal
Source: EMBO J. 2013 Jul 26;32(16):2231–47. doi: 10.1038/emboj.2013.161 (PMC3746198; doi:10.1038/emboj.2013.161)
Supplement: Source data for Figure 6 [file emboj2013161df6.pdf]

Figure 6

HA IP

Repeat 1 Repeat 2 Repeat 3

212-TS SQTY MNG SPTY SMSY SQQG- 234

M1	SSS>A	TS	AQTY	MNG	APTY	AMSY	SQQG
M2	TTS>A	TS	SQAY	MNG	SPAY	SMAY	SQQG
M3	YYY>A	TS	SQTA	MNG	SPTA	SMSA	SQQG
M4	Rep1>A	TS	AQAA	MNG	SPTY	SMSY	SQQG
M5	Rep2>A	TS	SQTY	MNG	APAA	SMSY	SQQG
M6	Rep3>A	TS	SQTY	MNG	SPTY	AMAA	SQQG
M7	Rep1+2>A	TS	AQAA	MNG	APAA	SMSY	SQQG
M8	Rep1+3>A	TS	AQAA	MNG	SPTY	AMAA	SQQG
M9	Rep2+3>A	TS	SQTY	MNG	APAA	AMAA	SQQG
M10	Rep1+2+3>A	TS	AQAA	MNG	APAA	AMAA	SQQG

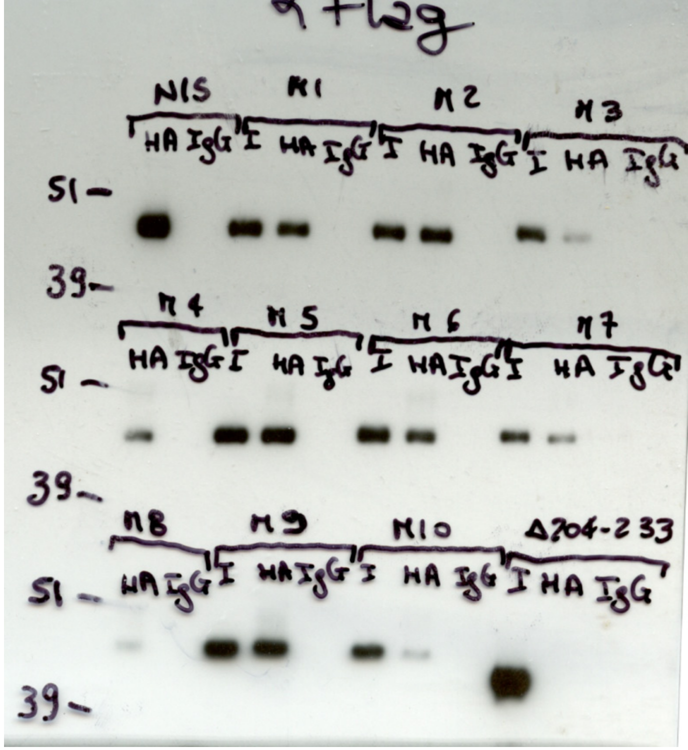

IB: α- Flag

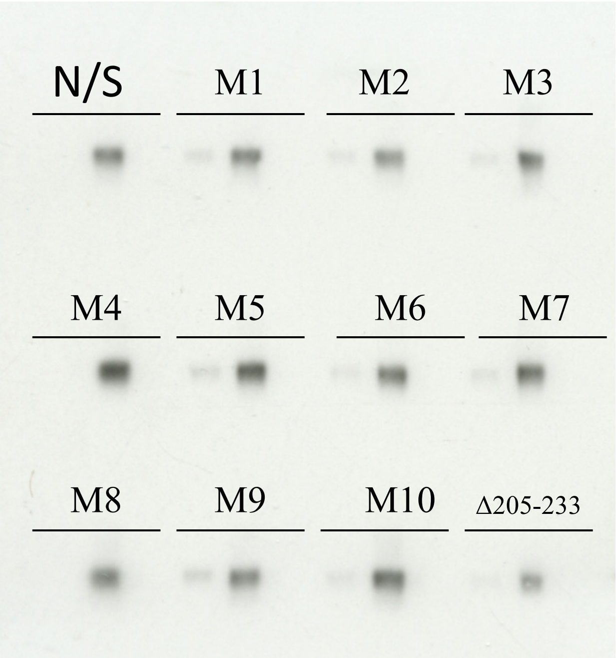

IB: α- HA

HA IP

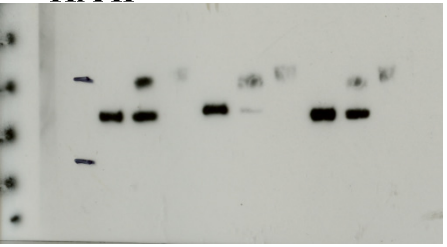

IB: α- Flag

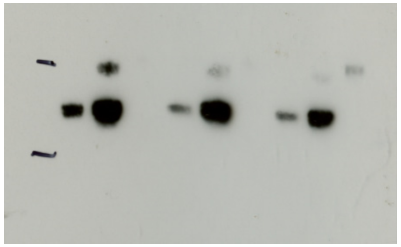

IB: α- HA

YYY>A	TS	SQTA	MNG	SPTA	SMSA	SQQG
YYY>F	TS	SQTF	MNG	SPTF	SMSF	SQQG
