# Supplementary material for: A direct physical interaction between Nanog and Sox2 regulates embryonic stem cell self-renewal
Source: EMBO J. 2013 Jul 26;32(16):2231–47. doi: 10.1038/emboj.2013.161 (PMC3746198; doi:10.1038/emboj.2013.161)

Figure 7A left

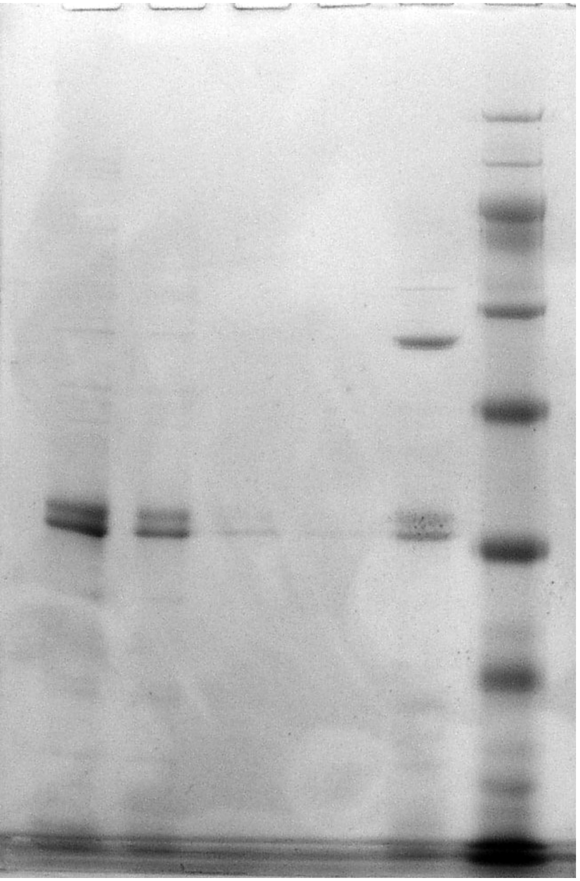

Figure 7A α-Nanog

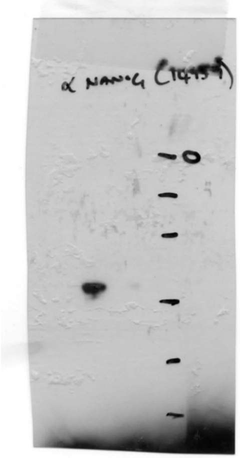

Figure 7A α-sox2

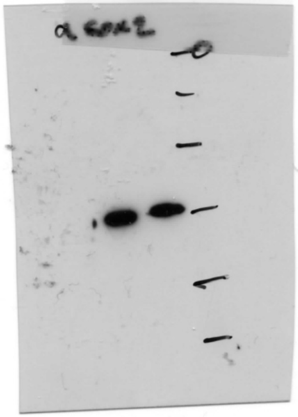

Figure 7B

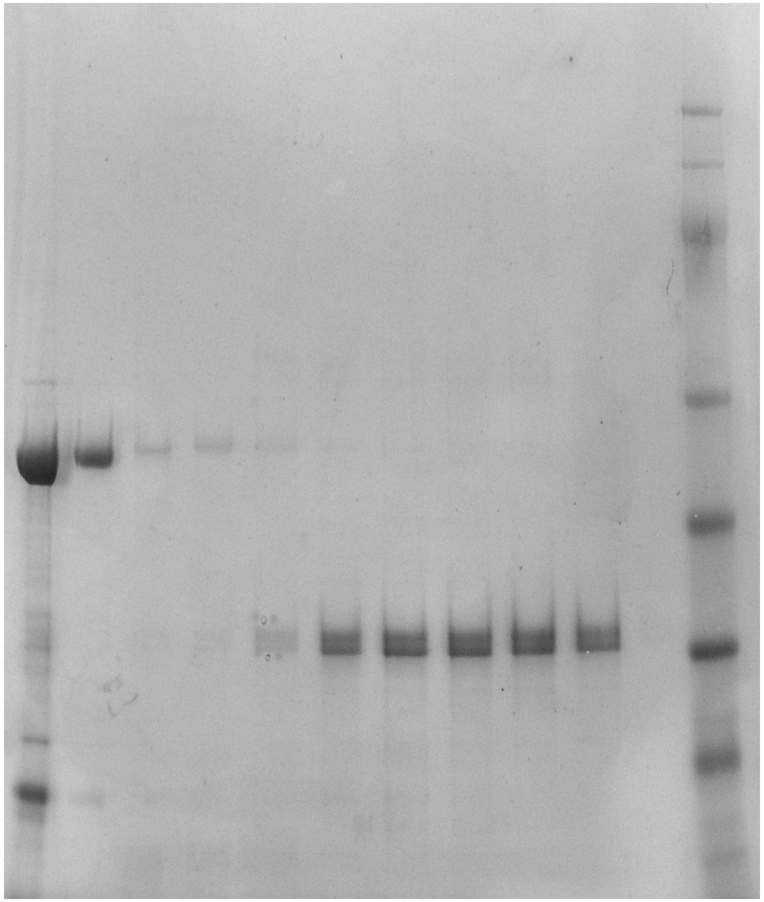

Supplement: Source data for Figure 7 [file emboj2013161df7.pdf]
